# Clinical and healthcare systems-level interventions for postpartum hemorrhage prevention and management in Nigeria: a systematic review of effectiveness and implementation outcomes

**DOI:** 10.3389/frhs.2026.1886853

**Published:** 2026-08-17

**Authors:** Melody Ucho, Princess Okelu, Sarim Faheem, Iretioluwa Adeola, Adwoa Ansong, Dorice Vieira, Nessa Ryan, Joyce Gyamfi, Emmanuel Peprah

**Affiliations:** 1 Department of Health Management and Systems Sciences, School of Public Health and Information Sciences, University of Louisville, Louisville, KY, United States; 2 Department of Global and Environmental Health, Implementing Sustainable Evidence-based interventions through Engagement (ISEE) Lab, New York University (NYU) School of Global Public Health, New York, NY, United States

**Keywords:** implementation science, low- and middle- income countries (LMIC), maternal mortality, Nigeria, obstetric hemorrhage, postpartum hemorrhage (PPH), PPH

## Abstract

**Background:**

Postpartum hemorrhage (PPH) remains the leading cause of maternal mortality globally and in Nigeria. Although clinical interventions may address PPH, less is known about their implementation within routine health systems. The objective of this systematic review was to synthesize the implementation research outcomes of interventions for PPH prevention and management in Nigeria for healthcare providers and patients.

**Methods:**

This systematic review of interventions for PPH in Nigeria searched seven electronic databases (i.e., Embase, Web of Science, MEDLINE, Global Health, Cochrane Library, PubMed, and PAIS) without date restrictions. Peer-reviewed studies reporting implementation and service outcomes, including adoption, sustainability, fidelity, and effectiveness, were included. Data were extracted on intervention type, setting, implementation, service and client outcomes. Risk of bias was assessed and visualized (via ROBVIS tool).

**Results:**

Of the 1,255 articles retrieved, 31 met the inclusion criteria, including 20 randomized controlled trials, eight observational, two mixed-methods, and one qualitative study. Most were conducted in tertiary facilities; none exclusively focused on primary-level care. Clinical interventions were mostly reported (*n* = 25). Evidence suggested comparable effectiveness between misoprostol and oxytocin for low-risk women. Device-based interventions, including the non-pneumatic anti-shock garment, were associated with improved stabilization and reduced mortality. Healthcare Systems-level interventions that include standardized care protocols in clinical management of PPH, particularly multicomponent bundled interventions such as the E-MOTIVE bundle and AMTSL, demonstrated high adoption and were associated with reductions in severe PPH. Implementation outcomes were infrequently reported (5 of 31 articles). Overall risk of bias was low-to-moderate.

**Conclusions:**

There was a notable gap in the evaluation and reporting of IS outcomes as well as the assessment of the patient's perspective from the receipt of interventions. Translation of effective PPH interventions may be constrained by limited implementation evaluation in Nigeria, particularly in primary-level care settings. Substantial lack of evidence exists in regions that bear a disproportionately high burden of PPH mortality.

**Systematic Review Registration:**

Open Science Framework (DOI 10.17605/OSF.IO/CYS2M).

## 1 Introduction

Postpartum hemorrhage (PPH) is a potentially life-threatening yet largely preventable obstetric complication that can occur during childbirth, immediately after delivery, or within the first weeks postpartum (1, 2). It is clinically defined as blood loss exceeding 500 mL within 24 h following vaginal delivery or more than 1,000 mL following cesarean birth (3–5). Recent revisions to the clinical definition have included the presentation with symptoms of hypovolemia within 24 h after the birth process (3). Uterine atony is the main cause of PPH, causing over 70% of PPH. Other causes include retained placenta, defective coagulation process, and trauma such as cesarean delivery (5). Despite the availability of proven preventive and therapeutic interventions, the World Health Organization (WHO) estimates that approximately 14 million women experience PPH each year, and the condition remains a leading cause of maternal mortality worldwide, contributing to the global maternal mortality rate of 197 per 100,000 live births in 2023 (6, 7). As a result, PPH prevention remains central to achieving Sustainable Development Goals (SDG) 2030 Target 3.1, which primarily aims to reduce the maternal mortality ratio to less than 70 per 100,000 live births by 2030 (8).

The burden of PPH is disproportionately concentrated in sub-Saharan Africa and South Asia, which together account for nearly 87% of global cases (6, 7). Additionally, in sub-Saharan Africa, PPH accounts for nearly 50% of maternal deaths, with maternal mortality ratios ranging from 500 to 1,000 per 100,000 live births in some settings (7, 9). Nigeria alone accounts for a disproportionate share of maternal deaths in Sub-Saharan Africa, ranking third by volume in sub-Saharan Africa after South Sudan and Chad, with PPH contributing substantially to this mortality burden (10–12). As the 2030 deadline for achieving SDG Target 3.1 approaches, Nigeria remains far from meeting this target. This persistent gap underscores the urgent need for scalable and context-appropriate interventions to reduce preventable maternal deaths.

In response to this global challenge, the WHO, the International Federation of Gynecology and Obstetrics (FIGO), and the International Confederation of Midwives (ICM) released updated guidelines in October 2025 to strengthen the prevention and management of PPH (8). These recommendations are operationalized through the E-MOTIVE bundle, a package of evidence-based interventions that includes early detection of excessive bleeding, uterine massage, administration of uterotonic agents, use of tranexamic acid to reduce hemorrhage, genital tract examination, and timely escalation of care when bleeding persists. However, translating these evidence-based recommendations into routine practice remains challenging in low-resource settings such as Nigeria. Structural and healthcare systemic barriers, including inadequate healthcare infrastructure, unreliable electricity supply, shortages of essential equipment and medications, and insufficient healthcare workforce capacity, limit the consistent implementation of recommended PPH interventions (13, 14). Structural constraints also influence the availability and timely use of specific clinical interventions. For example, tranexamic acid (TXA), a proven treatment for reducing mortality from PPH, is frequently unavailable or prohibitively expensive in many Nigerian facilities, sometimes requiring out-of-pocket purchase outside the hospital (16). In addition, restrictive clinical protocols and delays in emergency decision-making may further hinder rapid response during obstetric emergencies (4, 15).

Compounding these structural challenges, the burden of PPH in sub-Saharan Africa is further shaped by individual-level determinants that increase women's susceptibility to PPH at the point of delivery. Evidence from a systematic review and meta-analysis of PPH determinants across sub-Saharan Africa identified several key risk factors associated with increased PPH incidence, including multiparity, anemia, PPH and abortion history, multiple gestation, prior cesarean delivery, the absence of skilled birth attendance, and residence in rural areas (17). Critically, many of these individual risk factors cluster disproportionately among women with the least access to quality antenatal and intrapartum care. This inequity is evident in Nigeria's skilled birth attendance gap: recent estimates suggest that approximately 46% of births in Nigeria are attended by skilled providers (15), meaning that a substantial proportion of women — including those at the highest individual risk — deliver in community settings where timely detection and treatment of hemorrhage are not feasible.

While several alternative or complementary interventions have been proposed for use in low-resource settings, there remains limited evidence on which strategies are both clinically effective and feasible to implement at scale in Nigeria. Existing reviews on postpartum hemorrhage (PPH) prevention and management in Nigeria do not comprehensively synthesize the clinical effectiveness of a broad range of interventions and predominantly focus on provider-oriented implementation challenges. These reviews give limited attention to patient experiences and rarely integrate both patient and provider perspectives within a unified framework (18, 19). Importantly, adopting an evidence-based intervention does not guarantee its successful implementation or impact. Implementation science emphasizes the need to adapt interventions to local contexts, resources, and health system constraints (20, 21).

Guided by Proctor's implementation outcomes framework, which draws from the Institute of Medicine (IOM) standards of care (22, 23), this systematic review aims to generate context-specific evidence on the adoption, implementation, and effectiveness of PPH interventions in Nigeria. Specifically, this study has three objectives. First, it seeks to identify clinical interventions currently used to manage PPH among pregnant women in Nigeria. Second, it evaluates the implementation and service outcomes of these interventions including acceptability, appropriateness, sustainability, equity, and clinical effectiveness, from the perspectives of both healthcare providers and patients. Third, it aims to generate evidence-based recommendations to inform the scale-up of effective and sustainable PPH interventions within the Nigerian health system.

By examining the gap between clinical interventions and their real-world implementation, this review provides critical insights into how evidence-based practices can be adapted to resource-constrained settings. The findings are expected to inform policymakers, clinicians, and development partners in Nigeria and offer lessons for other low- and middle-income countries facing similar challenges. Ultimately, strengthening the implementation of effective PPH interventions is essential for reducing preventable maternal deaths and improving maternal health outcomes across sub-Saharan Africa.

## 2 Methods

This systematic review was guided by the PRISMA 2020 statement (24) and the review protocol was prospectively registered on Open Science Framework (DOI 10.17605/OSF.IO/CYS2M). The manuscript title was updated from the registered title to more precisely reflect the scope of the completed review; the protocol registration, inclusion criteria, and analytical framework remain consistent with the registered protocol.

### 2.1 Search strategy

In consultation with authors and a trained medical librarian (DV), the search strategy was developed. Seven databases, including PubMed, Medline (Ovid), Embase, Cochrane Library, Global Health, Web of Science, and Public Affairs Information Service (PAIS), were searched. The search was first conducted on February 13, 2025, and updated on March 10, 2025. There were no restrictions based on the year of article publication and the research methods employed. Published articles that were not written in the English language were excluded. The search did not include grey literature sources and articles from citation searching.

The following terms were used in the search:

(Nigeria (tw) OR Nigerian (tw) OR Abia (tw) OR Abia state (tw) OR Adamawa (tw) OR Adamawa state (tw) OR Akwa Ibom (tw) OR Akwa Ibom state (tw) OR Akwa-ibom (tw) OR Akwa-ibom state (tw) OR Akwa-Ibom (tw) Or Akwa-Ibom state (tw) OR Anambra (tw) OR Anambra state (tw) OR Bauchi (tw) OR Bauchi state (tw) OR Bayelsa (tw) OR Bayelsa state (tw) OR Benue (tw) OR Benue state (tw) OR Borno (tw) OR Borno state (tw) OR Cross river (tw) OR Cross river state (tw) OR Cross-river (tw) OR Cross-river state (tw) OR Cross-River (tw) OR Cross-River state (tw) OR Delta (tw) or Delta state (tw) OR Ebonyi (tw) OR Ebonyi state (tw) OR Edo (tw) OR Edo state (tw) OR Ekiti (tw) OR Ekiti state (tw) OR Enugu (tw) OR Enugu state (tw) OR Gombe (tw) OR Gombe state (tw) OR Imo (tw) OR Imo state (tw) OR Jigawa (tw) OR Jigawa state (tw) OR Kaduna (tw) OR Kaduna state (tw) OR Kano (tw) OR Kano state (tw) OR Katsina (tw) OR Katsina state (tw) OR Kebbi (tw) OR Kebbi state (tw) OR Kogi (tw) OR Kogi state (tw) OR Kwara (tw) OR Kwara state (tw) OR Lagos (tw) OR Lagos state (tw) OR Nasarawa (tw) OR Nasarawa state (tw) OR Niger (tw) OR Niger state (tw) OR Ogun (tw) OR Ogun state (tw) OR Ondo (tw) OR Ondo state (tw) OR Osun (tw) OR Osun state (tw) OR Oyo (tw) OR Oyo state (tw) OR Plateau (tw) OR Plateau state (tw) OR Rivers (tw) OR Rivers state (tw) OR Sokoto (tw) OR Sokoto state (tw) OR Taraba (tw) OR Taraba state (tw) OR Yobe (tw) OR Yobe state (tw) OR Zamfara (tw) OR Zamfara state (tw) OR Federal Capital Territory (tw) OR FCT (tw) OR Abuja (tw)) **AND** (health provider uptake (tw) OR health provider acceptance (tw) OR health care utilization (tw) OR healthcare utilization (tw) OR patient acceptance of healthcare (tw) OR patient attitude (tw) OR patient compliance (tw) OR patient participation (tw) OR patient adherence (tw) OR adoption (tw) OR adopt (tw) OR adopted (tw) OR adopts (tw) OR uptake (tw) OR utilization (tw) OR implementation (tw) OR “intention to try” (tw) OR appropriateness (tw) OR appropriate (tw) OR program evaluation (tw) OR perceived fit (tw) OR relevance (tw) OR compatibility (tw) OR suitability (tw) OR usefulness (tw) OR practicability (tw) OR program effectiveness (tw) OR cost (tw) OR costs (tw) OR cost effectiveness (tw) OR cost benefit (tw) OR cost analysis (tw) OR cost analyses (tw) OR affordability (tw) OR pricing (tw) OR cost measures (tw) OR cost comparison (tw) OR value (tw) OR expenditures (tw) OR feasibility (tw) OR feasible (tw) OR utility (tw) OR suitability (tw) OR practicability (tw) OR fidelity (tw) OR delivery (tw) OR adherence (tw) OR availability (tw) OR integrity (tw) OR quality (tw) OR “quality of medications” (tw) OR “quality of services” (tw) OR “quality of health services” (tw) OR “quality of medical service*” (tw) OR “quality of ante-natal services” (tw) OR “quality of antenatal service*” (tw) OR “quality of obstetric service*” (tw) OR reliability (tw) OR penetration (tw) OR institutionalization (tw) OR spread (tw) OR service access (tw) OR access (tw) OR accessible (tw) OR accessibility (tw) OR sustainability (tw) OR sustain (tw) OR sustainable (tw) OR program evaluation (tw) OR implementation (tw) OR evidence-based practice (tw) OR innovation (tw) OR scale (tw) OR assessment (tw) OR measurement (tw) OR translation research (tw) OR scale-up (tw) OR scalability (tw)) AND (prevention (tw) OR primary prevention (tw) OR primary disease prevention (tw) OR secondary prevention (tw) OR secondary disease prevention (tw) OR preventive management (tw) OR management (tw) OR preventive intervention* (tw) OR intervention (tw) OR treatment* (tw) OR protocol (tw) OR guideline* (tw) OR best practices (tw) OR practices (tw) OR care (tw) OR strategy (tw) OR obstetric service (tw) OR screening tool* (tw) OR bundle* (tw) OR obstetric hemorrhage bundle* OR OH bundle* (tw) OR therapy*(tw)) AND (obstetrical hemorrhage (tw) OR obstetric hemorrhage (tw) OR obstetric haemorrhage (tw) OR postpartum haemorrhage (tw) OR post-partum haemorrhage (tw) OR postpartum hemorrhage (tw) OR vaginal bleeding (tw) OR uterine hemorrhage (tw) OR uterine bleeding (tw) OR antepartum hemorrhage (tw) OR intrapartum hemorrhage (tw) OR maternal bleeding (tw) OR PPH (tw) OR antenatal bleeding (tw) OR ante-natal bleeding (tw) OR post-natal bleeding (tw) OR postnatal bleeding (tw)).

The search strategy presented above reflects the syntax used for PubMed, Medline (Ovid), Embase (Ovid), Cochrane Library (Ovid), Global Health (Ovid), Web of Science, and Public Affairs Information Service, PAIS (ProQuest). SR-Accelerator [recently upgraded to The Evidence Review Accelerator (TERA)], a web-based program that translates search syntax between databases, was used to translate search strategies on the Ovid, Web of Science, and ProQuest platforms. Broad hemorrhage-related terms were included to maximize search sensitivity; study eligibility required explicit focus on postpartum hemorrhage as defined by WHO criteria (i.e., blood loss > 500 mL within 24 h following vaginal delivery or >1,000 mL following cesarean section). Studies examining antepartum hemorrhage, intrapartum hemorrhage, or other bleeding conditions that did not occur in the postpartum phase and were not from the genital tract were excluded during the title/abstract screening phase.

Following the search, citations were uploaded to EndNote for data management and subsequently exported to Covidence, a systematic review management software.

### 2.2 Study selection and screening

Titles and abstracts of all retrieved articles were exported from EndNote into Covidence and independently screened by four reviewers (MU, PO, MA, and DV). Prior to formal screening, the reviewers piloted a small sample set of imported articles to establish a shared understanding of the eligibility criteria. Included articles were rated by at least two reviewers to determine eligibility for inclusion based on the study objective, and where consensus could not be reached, DV and MU jointly adjudicated unresolved disagreements to ensure that final inclusion and exclusion decisions were not determined by any single reviewer's judgment. Included articles were empirical studies; reviews were excluded. Using the population/patient, intervention, comparison, and outcome (PICO) framework (25), we developed eligibility criteria to include articles that assessed pregnant women or postpartum women receiving clinical interventions for PPH prevention or management, or healthcare providers involved in the prevention or management of PPH, and evaluated the use of clinical interventions for PPH within clinical settings in Nigeria. Furthermore, full texts of included articles were assessed for eligibility by three reviewers. Eligible studies were required to report implementation and/or service outcomes of PPH preventive clinical interventions, as defined by Proctor et al. (22) and IOM. Articles that did not meet the criteria described above were excluded. A third independent reviewer was assigned to resolve conflicts generated in the full-text screening phase.

### 2.3 Data extraction

A total of 1,255 articles were retrieved from the initial search. After duplicates were removed, 737 articles proceeded to the title and abstract screening phase. One hundred and thirty-four articles were included in the full text screening phase; of these, 103 articles that did not meet the eligibility criteria were excluded for various reasons, including wrong outcomes, incomplete study, wrong PPH outcomes, wrong study design, not a clinical setting, wrong clinical setting, wrong study population, full-text not available, not an intervention study, and studies not conducted in Nigeria (Figure 6). A total of 31 articles were included in the final data extraction phase. Data were extracted by at least two reviewers (PO, MA, AA, SF, DV), and MU facilitated consensus during the extraction phase. Data extraction was completed in Covidence using a template developed by the authors, which extracted contents of included articles into 3 tables as follows: (i) Study characteristics, (ii) PPH variables of interest, (iii) PPH outcomes. This format captured study, facility, and population-specific characteristics such as study design, sample size, geopolitical zone where the studied facility is located, cadre of attending health workers, mode of delivery, PPH type, type of intervention administered, timing of administration, implementation, and service outcomes measured, and key findings. Bundled strategies for PPH prevention and management are a set of interventions that may include clinical interventions adopted system-wide as protocols, as well as non-clinical interventions, such as provider training.

### 2.4 Inter-rater reliability

Inter-rater reliability for both title/abstract and full-text screening was assessed using Cohen's kappa (*κ*) statistic, providing a quantitative measure of agreement beyond chance. Kappa values were interpreted using established benchmarks, with higher values indicating greater inter-reviewer consistency and the overall reliability of the study selection process.

### 2.5 Risk of bias assessment

**Figure 1:**
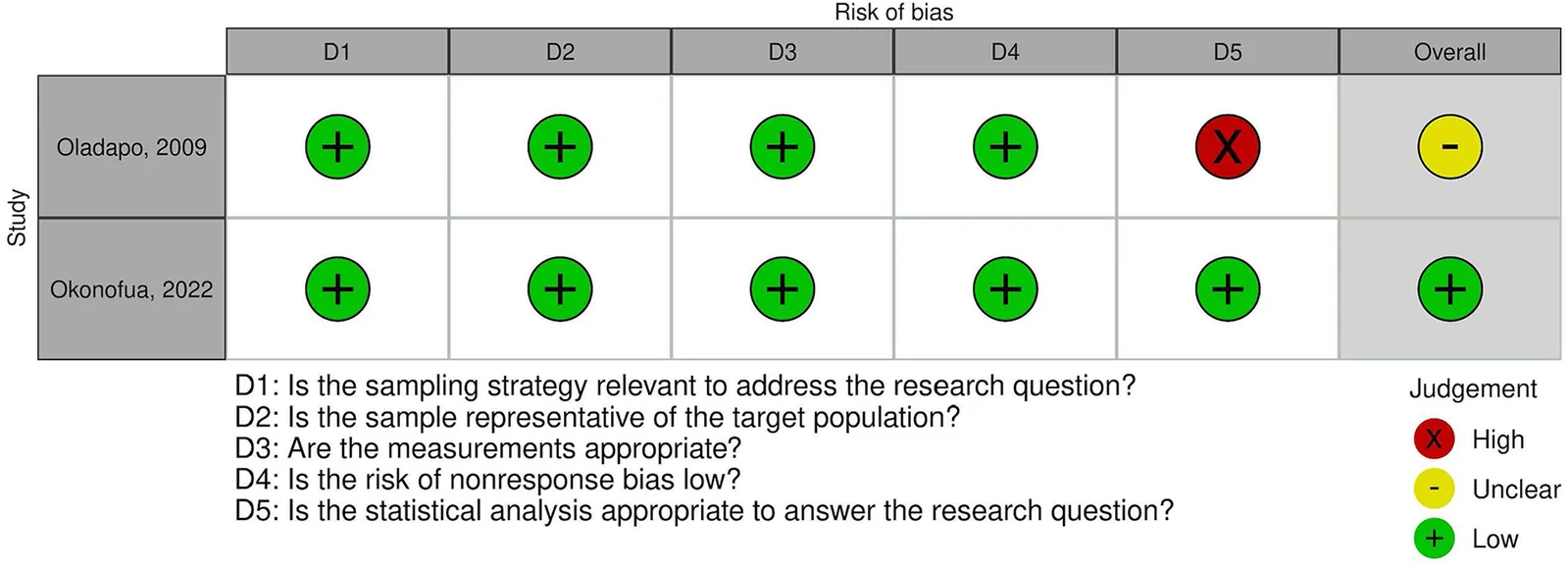
Risk of bias - quantitative descriptive.

**Figure 2:**
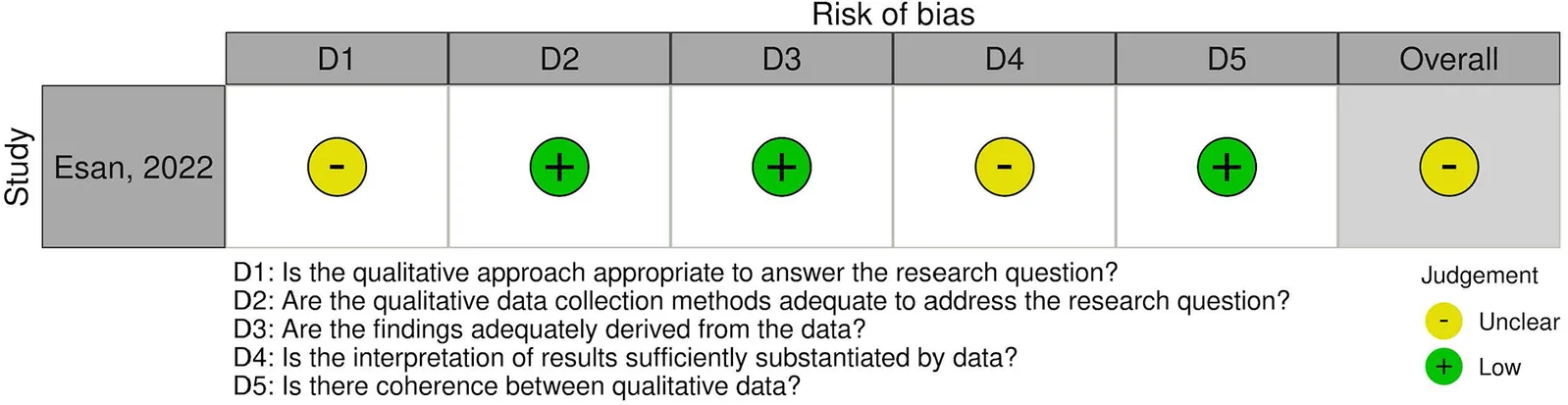
Risk of bias - qualitative studies.

**Figure 3:**
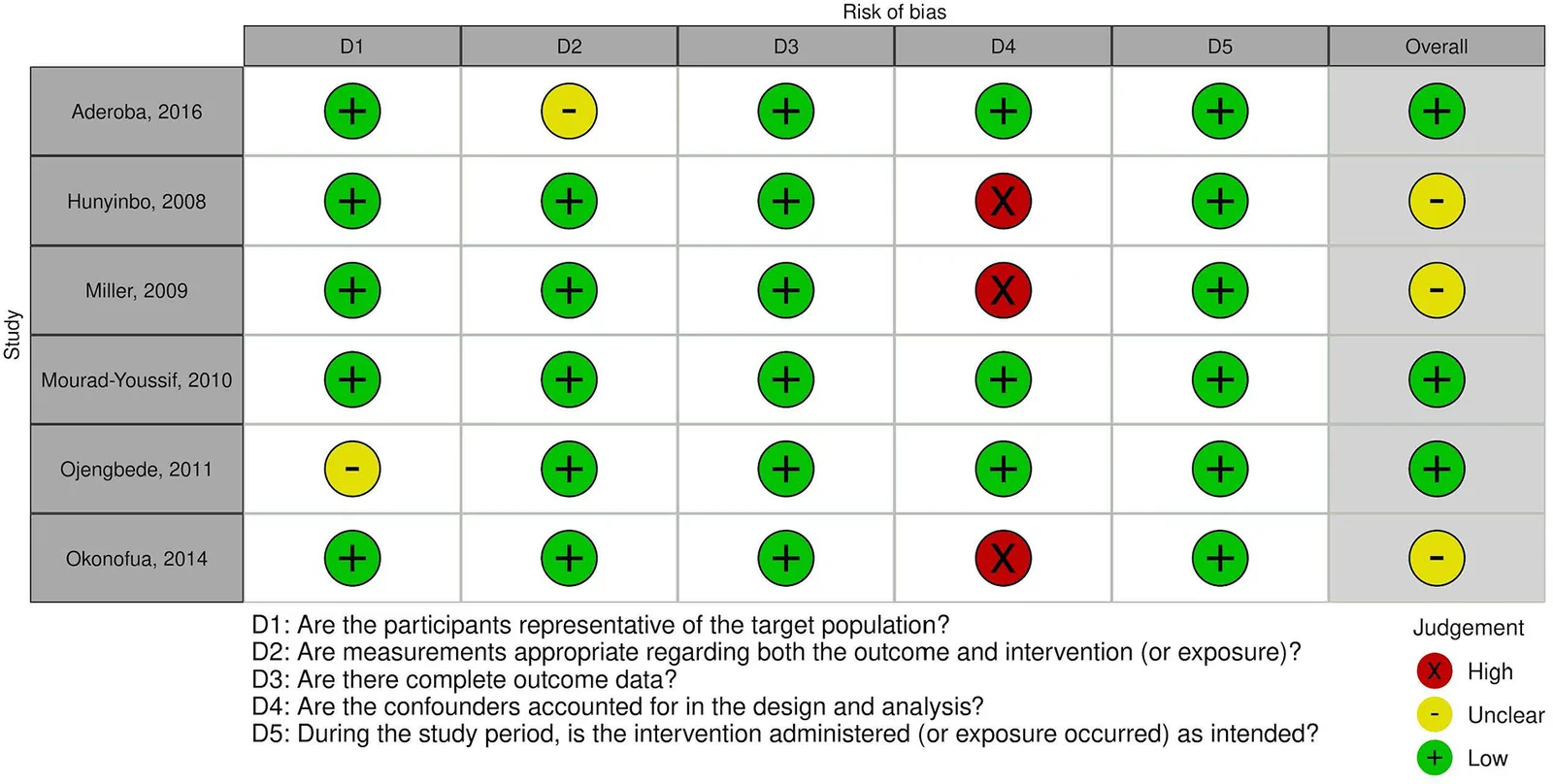
Risk of bias - non-RCTs.

**Figure 4:**
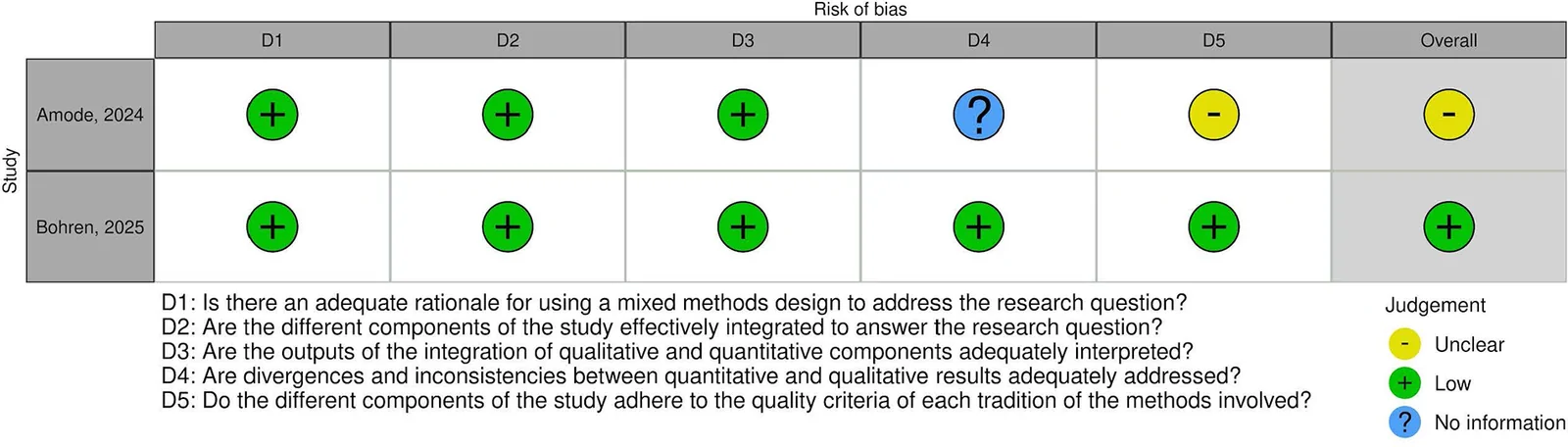
Risk of bias - mixed methods.

**Figure 5:**
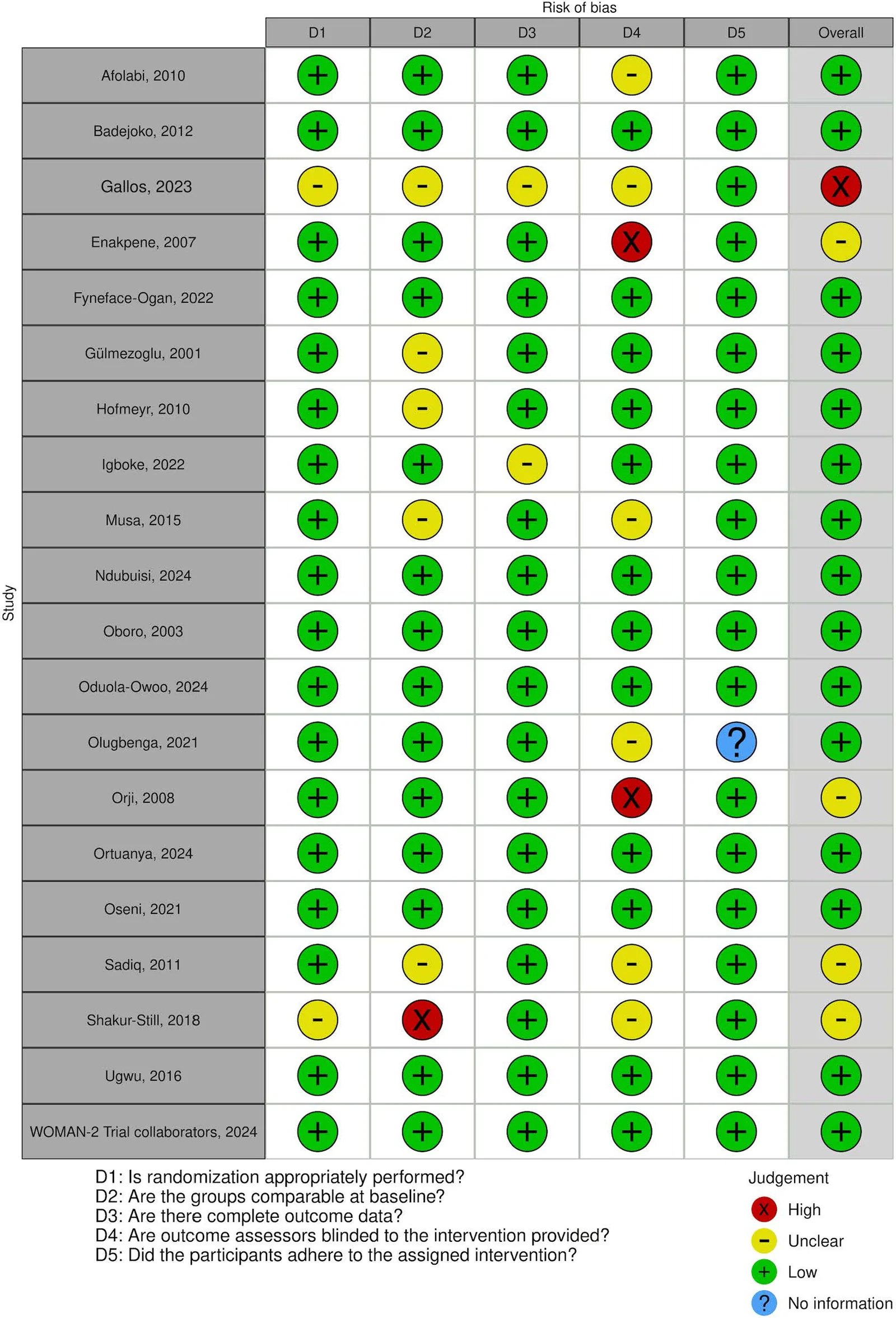
Risk of bias - RCTs.

Risk of bias was assessed using a validated tool (ASSESS) developed by Ryan et al. (26) and implemented via an accessible Google Form. The ASSESS tool was selected over design-specific tools such as RoB 2 or ROBINS-I because it was developed to evaluate implementation research outcomes across heterogeneous study designs within a single appraisal framework, consistent with the implementation science focus of this review. Future reviews focused exclusively on clinical effectiveness of RCTs may benefit from RoB 2. Included studies were appraised using standardized, study design–specific criteria to evaluate methodological quality and potential sources of bias. Study quality was subsequently visualized using the Risk of Bias Visualization (ROBVIS) tool (27), with scores assigned based on the extent to which each study met predefined criteria relevant to its design (Figures 1–5). Risk of bias was categorized as low, unclear, or insufficient information. Studies rated as unclear or lacking sufficient information were classified as having a high risk of bias.

## 3 Results

### 3.1 Study characteristics and geographic distribution

**Figure 6:**
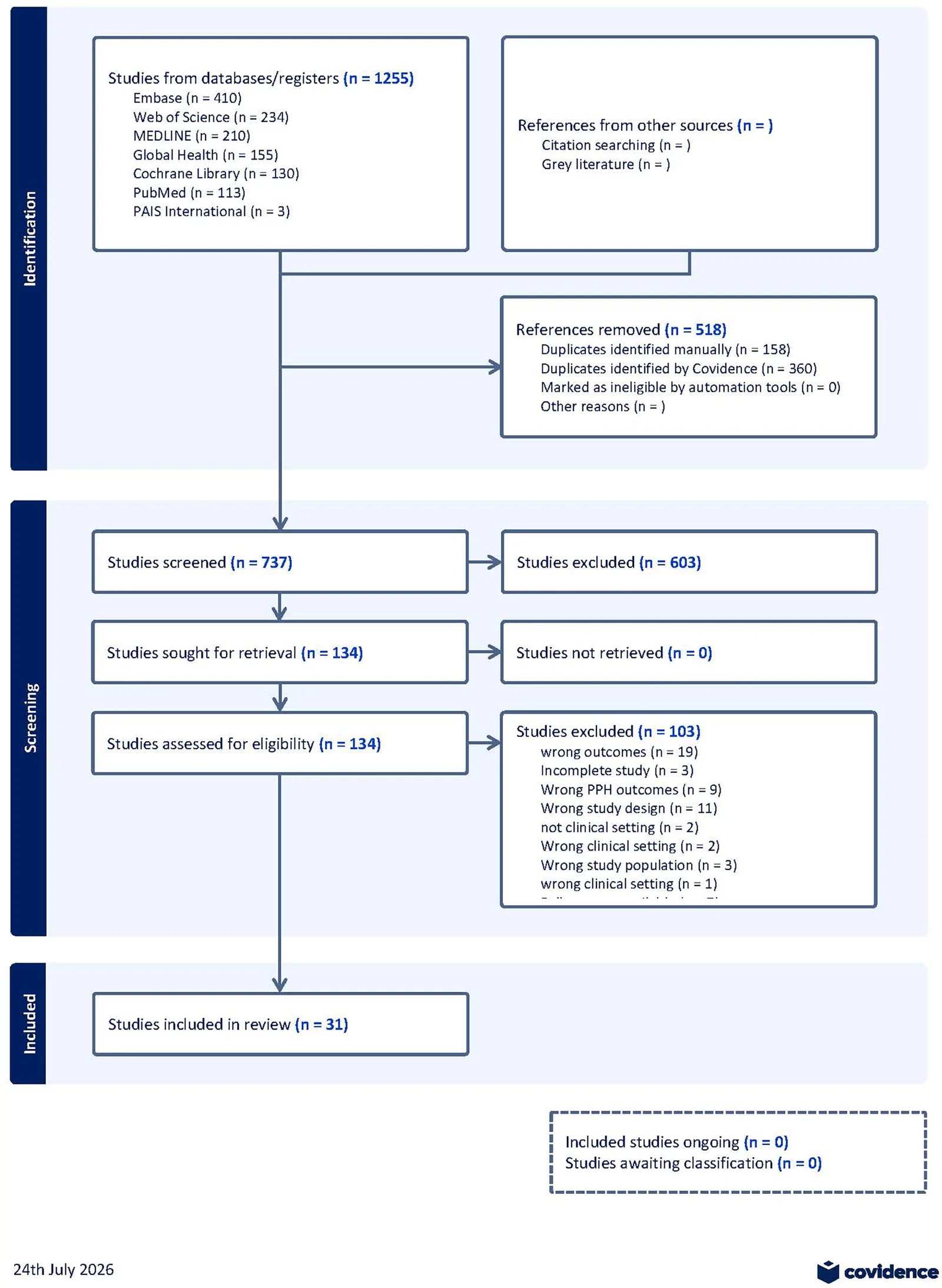
PRISMA flow diagram.

In our review, 1,255 articles were retrieved, 31 were included for extraction, and they were published between the years 2001 and 2024 (Figure 6). Of those, 20 were randomized controlled trials (RCTs), eight were observational studies, two were mixed-method designs, and one used a qualitative approach. Among the eight observational studies, six were cohort studies, one was a cross-sectional study, and one was a case-control study. Sample sizes varied across study designs (Tables 1, 2). RCTs generally included at least approximately 80 participants, whereas non-RCT and observational studies typically enrolled 130 participants or more. The geographic distribution of studies spanned Nigeria's six geopolitical zones. The majority were conducted in the South-West (*n* = 15) (28–42), followed by the South-South (*n* = 4) (43–46), North-West (*n* = 4) (32, 42, 47, 48), and North-Central (*n* = 4) (32, 42, 43, 49). Fewer studies were conducted in the South-East (*n* = 3) (50–52), and only one study was conducted in the North-East (*n* = 1) (53). Five studies were multi-country investigations (36, 54–57) and three were multiregional within Nigeria (32, 42, 43). One study did not specify its geographic location (58).

**Table 1:** Characteristics of studies assessing implementation outcomes.

Author (Year)	Study design	Sample size/population	Intervention	Comparator	Implementation outcome	Result
Amode, (42)	Mixed Methods	177 HCPs	Heat-stable Carbetocin, Misoprostol, and Oxytocin	Not specified	Acceptability among HCPs cost and safety	76% of the HCPs preferred HSC for PPH prevention, and 85% attributed their preference to HSC's effectiveness
						43% HCPs did not consider cost of uteronics as a factor impacting their use
Bohren, (54)	Mixed Methods	2,578 HCPs	E-MOTIVE bundle	Not specified	Fidelity, Adoption, Acceptability, and Feasibility	95.7% accepted use of the drape component; 99% adopted use of the bundle
Esan, (37)	Qualitative	15 HCPs	PPH management practices (oxytocin, uterine stimulation)	Not applicable	Appropriateness	Use of uterotonics and the stimulation of contractions listed by health workers as most appropriate
Hunyinbo (35)	Prospective cohort	HCP provision of care to 65 patients (phase 1) and 65 patients (phase 2)	Criteria-based audit of obstetric care	Baseline (phase 1)	Feasibility	Significant improvements in OH care (61% vs81%); the criteria-based audit was feasible
Oladapo, (34)	Observational (Cross-sectional)	HCP provision of care to 486 patients delivering vaginally	Active Management of Third Stage of Labour (AMTSL)	Not applicable	Adoption	AMTSL use varied by definition; positive impact on third-stage outcomes

IS, implementation science; HCP, healthcare provider; HSC, heat-stable carbetocin; PPH, postpartum hemorrhage; OH, obstetric hemorrhage. Results were restricted to findings aligning with the define implementation outcomes defined by Proctor et al.

**Table 2a:** Characteristics of studies assessing *effectivenes*s as A service outcome.

Author (Year)	Study design	Sample size/population	Intervention	Comparator	Result
Aderoba, (40)	Prospective observational	87 women who delivered vaginally or via C-section and had PPH that was not amenable to first-line therapy	Condom-catheter uterine balloon tamponade	Not specified	Controlled PPH in 89% of cases with lower morbidity
Amode, (42)	Mixed methods	177 HCPs; 18,480 deliveries	Heat-stable carbetocin, oxytocin, misoprostol administered at the third stage of labor	Not specified	76% preferred HSC; 94% reported it was effective in preventing blood loss
Badejoko, (31)	RCT	104 women undergoing vaginal delivery and had at least one PPH risk factor	Adjunctive rectal Misoprostol (600 µg) administered after routine AMTSL	Oxytocin 20 IU IV administered after routine AMTSL	No significant difference in blood loss or uterine atony
Bohren, (54)	Mixed methods	461 women delivering vaginally; 2,578 HCWs	E-MOTIVE bundle administered within 15 min of PPH diagnosis	Not specified	Effective for early detection of PPH
Gallos, (55)	RCT	17,300 (interventio); 20,909 (control)—vaginal delivery	E-MOTIVE bundle (started with monitoring trigger lines at 300 and 500 mL of collected blood within the first hour of birth)	Usual care	60% lower risk of severe PPH, laparotomy, or maternal death
Enakpene, (38)	RCT	200 women delivering vaginally	Misoprostol 600 µg oral administered when delivery was imminent (at the delivery of the anterior shoulder)	Methylergometrine 0.2 mg IM administered when delivery was imminent (at the delivery of the anterior shoulder)	Misoprostol is more effective in reducing blood loss
Gülmezoglu, (57)	RCT	9,348 women delivering vaginally	Misoprostol 600 µg oral administered immediately after delivery	Oxytocin administered immediately after delivery	A higher proportion had blood loss ≥ 1,000 mL with misoprostol
Hofmeyr, (36)	RCT	978 women delivering vaginally	Misoprostol 400 µg sublingual + oxytocics (ergometrine and oxytocin) administered immediately after delivery	Placebo administered immediately after delivery	No significant difference in blood loss
Igboke, (51)	RCT	170 women delivering vaginally	Tranexamic acid (TXA) 1 g IV within 2 min after delivery	Placebo within 2 min after delivery	Reduced mean blood loss (174.87 mL vs 341.07 mL)
Miller, (48)	Cohort	1,041 women with estimated initial blood loss of at least 750 mL, and at least 1 sign of shock	Non-pneumatic anti-shock garment (NASG) on study entry or as soon as the patient presents	Pre-intervention phase	NASG significantly reduced blood loss
Musa, (49)	RCT	140 women with low PPH risk delivering vaginally	Misoprostol 600 µg oral within 1 min of delivery and cord clamping	Oxytocin 10 IU IM within 1 min of delivery and cord clamping	Similar efficacy for PPH prevention
Ndubuisi, (50)	RCT	284 women delivering via Cesarean section	Intraoperative TXA 1 g IV + post-delivery oxytocin	Placebo + post-delivery oxytocin	Lowered mean blood loss (435.9 mL vs 918 mL)
Oboro, (45)	RCT	1,200 women delivering vaginally	Misoprostol 400 µg oral after delivery (within the third stage of labor)	Oxytocin 10 IU IM after delivery (within the third stage of labor)	Misoprostol can replace oxytocin in low-risk women
Oduola-Owoo, (29)	RCT	130 women scheduled for elective or emergency Cesarean section	TXA 1 g IV within 3 min after delivery of fetus following routine 100 µg Carbetocin or 10IU oxytocin at delivery	Placebo	Mean blood loss is significantly lower with TXA
Ogan, (44)	RCT	80 women undergoing Cesarean delivery	Carbetocin 100 µg IV administered after delivery of anterior shoulder	Oxytocin 2.5 (IU) IV + 5 (IU) saline infusion administered after delivery of anterior shoulder	Lower blood loss with carbetocin (280.23 mL vs 745.22 mL)
Ojengbede, (58)	Prospective cohort	1,296 women with estimated initial blood loss of at least 750 mL, and at least 1 sign of shock	NASG (applied at time of study admission)	Historical controls	Reduced PPH mortality in referral facilities
Okonofua, (32)	Prospective cohort	978 women with PPH due to uterine atony and delivering vaginally	Misoprostol 800 µg sublingual	Not specified	Reduced blood loss; not effective for all PPH forms
Okonofua, (43)	Case–control	18,181 women delivering both vaginally and via Cesarean section	Multifaceted health system intervention	Pre-intervention period	Lower PPH incidence over 21 months
Olugbenga, (46)	RCT	304 women admitted for emergency or elective cesarean section (not category 1)	TXA 10 mL preoperative	Placebo	Reduced mean blood loss (502.84 mL vs 699.46 mL)
Orji, (33)	RCT	200 women delivering vaginally	Oxytocin 10 IU IV after delivery of anterior shoulder	Ergometrine 0.5 mg IV after delivery of anterior shoulder	A slightly higher risk for postpartum hemorrhage, need for additional use of oxytocin, retained placenta, and manual removal of placenta in the ergometrine group compared with the oxytocin group. The difference was not significant
Ortuanya, (52)	RCT	660 women delivering via Cesarean section	TXA 1 g IV at least 10 min prior to the onset of surgery	Placebo	lower mean blood loss (442.94 ± 200.97 versus 801.28 ± 258.68 mL; *p* = 0.001), significantly lower incidence of postpartum hemorrhage and lower need for use of additional uterotonic agents
Oseni, (47)	RCT	390 women delivering via Cesarean section	TXA 1 g + oxytocin administered at least 5 min prior to surgery	Oxytocin alone	Significantly reduced blood loss
Shakur-Still, (28)	RCT	20,021 women delivery either vaginally or via Cesarean section	TXA 1 g IV administered after delivery (immediate postpartum)	Placebo	Inhibited fibrinolysis associated with PPH
Ugwu, (30)	RCT	124 women delivering vaginally	Misoprostol 200 µg vs 400 µg administered after delivery of anterior shoulder	Dose comparison	Similar blood loss; fewer side effects at 200 µg
Uthman, (53)	RCT	300 women delivering vaginally	Misoprostol 600 µg oral within 3 min after delivery	Oxytocin 10 IU IV at the delivery of the baby's shoulder	Better PPH prevention with misoprostol
WOMAN-2, (41)	RCT	30,662 women who delivered vaginally	TXA 1 g IV within 15 min of cord clamping or cutting	Placebo	No reduction in PPH risk in anaemic women
Youssif, (56)	Cohort	854 women with estimated initial blood loss of at least 750 mL, and at least 1 sign of shock	NASG was applied at the time of admission	No NASG	Lowers mortality and intervention buys time for definitive treatment

IS, implementation acience; HCP, healthcare provider; HSC, heat-stable carbetocin; PPH, postpartum hemorrhage; OH, obstetric hemorrhage. Results were restricted to findings aligning with IOM definitions of service outcomes.

**Table 2b:** Characteristics of studies assessing other service outcomes.

Author (Year)	Study design	Sample size/population	Intervention	Comparator	Service outcome	Result
Afolabi, (39)	RCT	200 women who delivered vaginally	Misoprostol 400 µg oral administered after cord clamping	Oxytocin 10 IU IM administered after cord clamping	Safety	Average blood loss is comparable; 4% nausea in the misoprostol group
Amode, (42)	Mixed methods	177 HCPs; 18,480 deliveries	Heat-stable carbetocin, oxytocin, misoprostol administered at the third stage of labor	Not specified	Effectiveness, Safety	99% reported HSC had a good safety profile; easy administration and fewer side effects
Hofmeyr, (36)	RCT	978 women delivering vaginally	Misoprostol 400 µg sublingual + oxytocics administered immediately after delivery	Placebo administered immediately after delivery	Safety, Effectiveness	No significant safety concerns identified
Ndubuisi, (50)	RCT	284 women delivering via Cesarean section	Intraoperative TXA 1 g IV + oxytocin post-delivery oxytocin	Placebo post-delivery oxytocin	Effectiveness, Safety	TXA is effective and safe; no significant adverse events
Okonofua, (61)	Prospective cohort	978 women with PPH due to uterine atony and delivering vaginally	Misoprostol 800 µg sublingual	Not specified	Effectiveness, Safety	Effective, but additional treatments are required for some PPH forms
Olugbenga, (46)	RCT	304 women admitted for emergency or elective cesarean section (not category 1)	TXA 10 mL preoperative	Placebo	Effectiveness, Safety	TXA is effective with no harm to newborns
Orji, (33)	RCT	200 women delivering vaginally	Oxytocin 10 IU IV after delivery of anterior shoulder	Ergometrine 0.5 mg IV after delivery of anterior shoulder	Effectiveness, Safety	Fewer side effects and reduced need for additional oxytocics compared with ergometrine
Ortuanya, (52)	RCT	660 women delivering via Cesarean section	TXA 1 g IV at least 10 min prior to the onset of surgery	Placebo	Effectiveness, Safety	No significant difference; similar safety profiles in both groups
Ugwu, (30)	RCT	124 women delivering vaginally	Misoprostol 200 µg vs 400 µg sublingual administered after delivery of anterior shoulder	Dose comparison	Safety, Effectiveness	200 µg had fewer adverse effects; both doses were equally effective
WOMAN-2, (41)	RCT	30,662 women who delivered vaginally	TXA 1 g IV within 15 min of cord clamping or cutting	Placebo	Safety, Effectiveness	TXA is safe, but did not reduce PPH in women with moderate to severe anemia

Studies were conducted across a range of facility types, including tertiary teaching and federal referral hospitals, as well as secondary-level general, specialist, and maternity hospitals. Most studies were implemented in tertiary-level facilities (*n* = 21), with fewer in secondary-level facilities (*n* = 6). Three studies spanned mixed facility settings spanning tertiary, secondary, and primary levels (41, 42, 53). No study was conducted exclusively in primary-level facilities, and one study did not specify the facility type.

### 3.2 Categories of interventions

The included studies evaluated a broad spectrum of clinical interventions for PPH prevention and management. These were categorized into three overarching categories consistent with the clinical application of interventions as operationalized across the 31 included studies: (a) pharmacological interventions for PPH prevention and management, including uterotonics and tranexamic acid; (b) rescue interventions, encompassing device-based approaches deployed in response to established severe hemorrhage; and (c) Healthcare systems-level interventions, focusing on service delivery, training, supportive supervision, and bundled interventions. Most studies (*n* = 25) assessed pharmacological interventions. The most frequently evaluated uterotonics were misoprostol (*n* = 11) and oxytocin (*n* = 10) (30–33, 37–39, 42, 44, 45, 47, 49, 50, 53, 57). Studies frequently evaluated effectiveness (which may refer to prevention and/or reduction in blood loss) and safety relative to placebo, compared agents head-to-head (most often misoprostol vs. oxytocin), or examined differences in dosage and routes of administration. Tranexamic acid (TXA) was evaluated in eight studies, typically as a 1 g dose, and compared with placebo. Less frequently studied uterotonics included carbetocin (*n* = 2) (42, 44), ergometrine (*n* = 2) (33, 36), and methylergometrine (*n* = 1) (38). Medical devices were evaluated in fewer studies. These included the non-pneumatic anti-shock garment (NASG) (*n* = 3) and condom-catheter uterine balloon tamponade (C-UBT) (*n* = 1) (40).

#### 3.2.1 Pharmacological interventions for PPH prevention and management

##### 3.2.1.1 Misoprostol and oxytocin

Evidence comparing misoprostol and oxytocin for PPH prevention and management was mixed but generally suggested comparable effectiveness. Two studies reported similar effectiveness between misoprostol and oxytocin when administered among women delivering vaginally (31, 49). However, other studies suggested that misoprostol may be most effective among women at a lower baseline risk of PPH (32, 45). When compared with ergometrine, misoprostol was associated with reductions in both the incidence of PPH and the proportion of women experiencing blood loss exceeding 500 mL (38). Evidence on adjunctive use was less favorable. Among women delivering vaginally and already receiving uterotonics such as oxytocin or ergometrine, the addition of misoprostol did not significantly reduce blood loss (36). Furthermore, when used as a sole prophylactic agent, a slightly higher proportion of women receiving misoprostol required additional uterotonics compared with those receiving oxytocin (57). Across all studies, misoprostol was consistently associated with transient, dose-dependent adverse effects, particularly pyrexia and shivering, usually one hour after administration. These side effects were less frequent at lower doses (e.g., 200 µg).

##### 3.2.1.2 Tranexamic acid (TXA)

Five studies (29, 46, 50–52) examined the use of TXA for PPH prevention and management among women delivering vaginally and women who underwent via cesarean section and found that TXA reduced blood loss in both cases, when administered preoperatively or 2–10 min post-delivery, compared to placebo or standard uterotonic care. However, findings from the WOMAN-2 trial indicated that prophylactic TXA administered 15 min after umbilical cord clamping did not significantly reduce blood loss. Evidence from surgical settings suggested a potential benefit when TXA was combined with oxytocin. In one study, the combination reduced intraoperative and postoperative blood loss (435.9 ± 34.0 vs. 918.0 ± 258.7) and postoperative blood loss (232.7 ± 67.4 vs. 717.0 ± 317.6) compared to oxytocin alone (50).

##### 3.2.1.3 Carbetocin

Carbetocin was evaluated in two studies (42, 44). In one randomized trial among women undergoing cesarean delivery, a substantially greater proportion of women receiving carbetocin experienced blood loss of less than 500 mL compared to those receiving oxytocin (72.5% vs. 37.5%) (44).

#### 3.2.2 Rescue interventions

Rescue interventions were device-based measures applied to stabilize women with established severe PPH and to control hemorrhage. They include Non-Pneumatic Shock Garment (NASG) and Condom-catheter tamponade (C-UBT). These were evaluated primarily in observational studies. NASG was generally used as a first-line stabilization measure for women with severe PPH, while C-UBT was employed as a second-line intervention when bleeding persisted (40). Both devices were reported to significantly improve hemodynamic stabilization and hemorrhage control (*P* < 0.0001) (40, 58). Across studies, their use was associated with reductions in PPH-related mortality among affected cases.

#### 3.2.3 Healthcare systems-level interventions

Healthcare Systems-level interventions were evaluated in six studies (34, 35, 37, 43, 54, 55). Five of the included studies (34, 35, 43, 54, 55) assessed multicomponent interventions and described a set of widely recommended or evidence-based clinical and/or non-clinical interventions. These interventions typically included staff training, protocol standardization (e.g., AMTSL), early warning systems, and rapid-response bundles such as the E-MOTIVE bundle. Multicomponent system interventions were generally associated with improved early detection and management of hemorrhage. Notably, the E-MOTIVE bundle was reported to reduce the incidence of severe PPH by approximately 60% (*P* < 0.001) (55).

### 3.3 Implementation science outcomes

Implementation outcomes were infrequently and inconsistently reported (Table 1). Twenty-six of the 31 articles did not specify implementation outcomes. Among the five studies that reported implementation outcomes (34, 35, 37, 42, 54), acceptability, and adoption were the most frequently reported (*n* = 3). Bohren et al. refer to *acceptability* as “the extent to which strategies are acceptable to health workers, encompassing their affective attitudes toward the intervention as well as their perceptions of its benefits, both of which may facilitate or hinder its implementation” (54). In contrast, Amode et al. conceptualized *acceptability* more quantitatively, defining it as the proportion of health providers who adopted the studied intervention for PPH prevention (42). Meanwhile, Oladapo et al. focused on the concept of *adoption*, describing it as the uptake, adherence, and extent to which health providers utilize a standardized practice or intervention (34). Multi-component interventions, particularly the E-MOTIVE bundle and AMTSL practices, demonstrated high levels of healthcare worker acceptance and adoption, with reported adoption rates approaching 99%. Within intervention groups demonstrating approximately 91% adherence to the bundle, PPH detection improved by up to 93% (54, 55).

*Appropriateness* and *feasibility* were reported in three studies (Table 1) (35, 37, 54). Esan et al. referred to *appropriateness* as “the perceived fit or relevance of the studied intervention to the given setting,” whereas Bohren et al. defined feasibility as “the facilitators and barriers to using the specified intervention within a given setting.” (37, 54). Hunyinbo et al. provided no operational definition for *feasibility*. *Fidelity*, reported by Bohren et al, was defined as “the degree to which interventions were used as intended.”^53^ Most outcomes were rarely examined, with only one study reporting the financial cost of implementation efforts. In the study that evaluated cost, the majority of health providers (43%) reported that the cost of a uterotonic did not influence their clinical decision or uterotonic preference (42). No studies reported outcomes related to *penetration* or *sustainability*. Among the five studies that reported implementation outcomes, two primarily examined healthcare worker populations (37, 42). Most implementation evaluations were conducted within multicomponent, systems-level interventions, including bundled interventions and care-delivery approaches such as the E-MOTIVE package, Active Management of the Third Stage of Labor (AMTSL), and maternal death surveillance and response systems. Pharmacological interventions were underrepresented in implementation analyses, with only one study examining implementation outcomes for uterotonics (misoprostol, oxytocin, and carbetocin) (42).

### 3.4 Service and client outcomes

Across the included studies, service outcomes were reported more frequently but remained limited in scope. *Effectiveness* was the most commonly reported outcome (*n* = 27) (Table 2a). Effectiveness was mostly measured as control or reduction in blood loss with the given intervention, either in early detection of PPH or in active reduction of the risk of uterine atony and resultant blood loss. Effectiveness was mostly measured from the perspectives of healthcare providers or through observations of operations in the delivery ward. The next most frequently reported outcome was *safety* (*n* = 10), mostly reported as fewer adverse effects from the use or implementation of an intervention. Examples of adverse effects reported include nausea and fever observed among the mothers, and any side effects on the newborn following the use of the intervention. No studies were explicit in evaluation and reporting of *patient-centeredness, patient-satisfaction, equity, timeliness, and efficiency as defined in the IOM guideline.* In addition, while three studies (37, 42, 54) provided provider perspectives on the use of interventions, none of the included studies provided patient perspectives.

### 3.5 Risk of bias (ROB) assessment

The Risk of Bias Assessment was assessed for all 31 included studies using the design-specific criteria and guidelines developed by Ryan et al. (26), and visualized using the Risk of Bias Visualization (ROBVIS) tool developed by McGuinness et al. (27). Overall, the findings indicated a predominantly low-to-moderate risk of bias across all included studies (Figures 1–5). Among the 20 RCT, methodological quality was generally robust, with the primary limitation being inadequate blinding of outcome assessors. Mixed-method (*n* = 2), observational (*n* = 2), and qualitative (*n* = 1) studies highlighted greater variability in methodological rigor, sampling, and analytical quality. Observational and longitudinal studies raised concerns about confounding and selection bias, while qualitative studies highlighted potential limitations in sampling and analytical transparency. The overall body of evidence demonstrates acceptable methodological quality; non-randomized designs were more susceptible to bias, particularly with respect to confounding and selection processes.

### 3.6 Inter-rater reliability (IRR)

Inter-rater reliability across reviewers ranged from fair to moderate agreement during both title/abstract and full-text screening, with the highest agreement observed to be *κ* = 0.50 for title/abstract screening and *κ* = 0.56 for full-text review (Supplementary Table S1). Although some reviewer pairs demonstrated low or null kappa values, these findings should be interpreted cautiously, as several comparisons were based on very small sample sizes. The most meaningful estimates of agreement are those from the primary reviewer pair (MU and PO), who independently screened the largest volume of articles across both the title/abstract and full-text phases, returning kappa values of *κ* = 0.50 and *κ* = 0.56 respectively, alongside proportionate agreement of 0.82 and 0.80 reflecting fair-to-moderate agreement. Low and null kappa values observed in other reviewer pairs are attributable to the statistical limitations of the kappa coefficient at very small sample sizes, as these pairs screened batches of as few as two to ten articles; at such scales, kappa is well-established to be unstable and unreliable as a measure of agreement. The NaN values observed for two reviewer pairs at full-text screening denote mathematically undefined kappa arising from complete agreement on exclusion across all articles in those batches, reflecting consensus not disagreement. In addition, given that several reviewer pairs demonstrated a high proportionate agreement alongside low kappa values may reflect the “kappa paradox,” where high agreement on exclusion decisions increases the probability of chance agreement and lowers the kappa statistic.

## 4 Discussion

This review identifies clinical interventions implemented to prevent PPH in Nigeria and synthesizes reported implementation and service outcomes. Although a wide range of pharmacologic, device-based, and healthcare systems-level interventions demonstrated clinical effectiveness to prevent and manage PPH and its health consequences, substantial gaps remain in geographic representation, primary care evidence, and implementation science evaluation. Similar reviews emphasize that while the availability of interventions are crucial for reducing PPH mortality in low- and- middle- income countries, they may present little effects when not contextually implemented (59).

First, only five of the 31 studies explicitly evaluated and reported implementation research outcomes as delineated by Proctor et al. and the IOM guideline, and even among these, reporting was limited in scope. Most implementation research focused on healthcare workers’ perceptions and adoption of uterotonics or bundled care protocols. There was minimal exploration of women's perspectives, despite the centrality of patient experience to maternal health care. One of the included studies examined the influence of uterotonic cost on provider preference and found that most providers did not consider the cost of uterotonics to be a major determinant in clinical decision-making during patient care (42). In addition, no studies evaluated the efficiency of interventions, which represents optimization of effective interventions at minimal cost. While the disregard of the cost of uterotonics in PPH management may reflect adherence to clinical standards that prioritize effective treatment, it also underscores the potential financial burden placed on patients, particularly in settings such as Nigeria, where healthcare is largely financed through high out-of-pocket expenditures (60). Furthermore, understanding women's perceptions of uterotonics, device-based interventions, or bundled protocols—including issues of trust, informed consent, side-effect tolerance, and cost of care remains an important but underexplored dimension of PPH prevention. While extant literature provides qualitative insight from patient perspectives into barriers related to access to care, such as a lack of personalized interaction with providers, extended wait times, and transportation (61, 62), quality and cost components are critical components for effective implementation of PPH interventions.

Second, none of the included studies specifically investigated PPH prevention in primary-level health facilities. Given that primary healthcare centers are often the first, and sometimes only, point of contact for pregnant women, particularly in rural areas (63), this gap is consequential. While this gap could be explained by the increased likelihood of referral of high-risk pregnancies to secondary and tertiary facilities, multicomponent interventions such as criteria-based protocols may be useful in primary-care settings. Studies that aggregated data from tertiary, secondary, and primary facilities may obscure the magnitude in staffing capacity, infrastructure, referral systems, and supply-chain reliability (64, 65). Primary care facilities frequently rely on task-shifting strategies and are staffed primarily by nurses, midwives, and community health extension workers rather than physicians. Without evidence tailored to these contexts, intervention recommendations may not fully account for operational constraints. The absence of primary-level evidence in this review suggests that strengthening quality improvement capacity and implementation research at the primary-care level may be an important step toward improving maternal outcomes at scale.

A third notable gap relates to the geographic distribution of studies, which reveals important inequities in research production. There was a notable paucity of studies originating from North-Eastern Nigeria, a region that bears a disproportionately high burden of maternal mortality attributable to PPH (11, 66–68). The limited representation of this region restricts context-specific understanding of how PPH interventions perform in settings characterized by insecurity, workforce shortages, and fragile health systems (69, 70). In addition, the underrepresentation of North-Eastern Nigeria in the available evidence base points to an urgent need to generate locally grounded evidence in such high-burden settings, as equitable progress in maternal health is unlikely without it (Tables 1, 2a, and 2b).

Despite these gaps, this review demonstrated that healthcare workers in Nigeria may be generally receptive to adopting structured, comprehensive PPH protocols. Multicomponent bundles, particularly the E-MOTIVE package and AMTSL, showed high levels of acceptance and adoption in the settings they were implemented (e.g., secondary and tertiary-level facilities (34, 54). Evidence suggests that this receptivity may be closely linked to structured and repeated training (54). For example, implementation of the E-MOTIVE bundle was preceded by intensive training sessions led by external facilitators, followed by refresher sessions within three months. Similar training components were reported in other bundled interventions. These findings align with broader LMIC evidence indicating that HCWs are more motivated to adopt interventions when they address real-world clinical challenges and when sufficient training ensures confidence and self-efficacy (71). Conversely, variability in AMTSL definitions and fidelity to the AMTSL protocol across different settings, as well as a lack of standardized training, have previously been shown to undermine fidelity and effectiveness (34). Together, these findings reinforce that clinical effectiveness alone is insufficient; structured training, standardization, and supportive supervision are essential to successful implementation.

Regarding pharmacologic interventions, the evidence suggests that misoprostol should be used in a context-specific manner. Misoprostol demonstrated comparable effectiveness to oxytocin in several studies and outperformed ergometrine in reducing moderate blood loss among lower-risk women (32, 38, 53, 57). However, its side-effect profile, particularly dose-dependent pyrexia and shivering, must be considered. When used as an adjunct to oxytocin, misoprostol did not consistently confer additional benefit sufficient to offset these adverse effects (31). Evidence from outside Nigeria suggests that combination therapy may provide benefit in certain contexts, but effectiveness appears to diminish among women at the highest risk of severe PPH (72). In low-resource environments where cold-chain storage for oxytocin cannot be guaranteed, heat-stable misoprostol may present an important alternative.

TXA demonstrated effectiveness in several clinical scenarios, particularly when used preoperatively or within 2–10 min following cesarean delivery. However, prophylactic administration among anemic women post-delivery did not significantly reduce blood loss in the WOMAN-2 Trial (41). One plausible explanation relates to the role of red blood cells (RBCs) in clot formation. RBCs contribute to clot structure and stability, enhancing antifibrinolytic efficacy. In anemic women, reduced RBC availability may compromise clot integrity, thereby attenuating the effectiveness of TXA (73). These findings highlight a broader systems-level implication: preventing and treating maternal anemia during pregnancy may be as important for PPH prevention as intrapartum pharmacologic strategies (74). Strengthening antenatal anemia screening, iron supplementation, and nutritional interventions should therefore be considered integral to comprehensive PPH risk reduction (75).

Evidence on carbetocin remains limited but promising. One trial suggested superior effectiveness compared to oxytocin in cesarean deliveries (44). Evidence from a meta-analysis also suggested similar effectiveness when carbetocin is administered to women undergoing vaginal delivery (76). However, differences in delivery modes and contextual factors, such as cold-chain challenges that affect oxytocin stability, complicate comparisons with broader meta-analytic findings. Carbetocin's heat stability may offer logistical advantages in settings with unreliable electricity, though cost considerations remain relevant (77).

Combination pharmacologic therapies, such as TXA plus oxytocin, demonstrated potential in reducing intraoperative and postoperative blood loss. However, in resource-constrained settings, prioritizing single interventions of comparable efficacy may be more cost-effective and operationally feasible. Device-based interventions such as the C-UBT and the NASG offer additional pragmatic alternatives. The reusability of NASG may confer cost-effectiveness in low-resource contexts. Importantly, none of the reviewed interventions were associated with major safety concerns, reinforcing their potential applicability across diverse care settings.

### 4.1 Policy implications

Reducing maternal mortality from PPH in resource-limited settings such as Nigeria requires more than clinical interventions alone. Recent evidence further underscores that addressing PPH within broader multidisciplinary frameworks and locally adaptable strategies is essential for reducing maternal mortality in resource-limited settings (81). The absence of evidence from primary care in this review suggests that strengthening quality improvement capacity and implementation research at the primary-care level, including equipping primary centers with essential uterotonics, emergency devices, and standardized protocols while reinforcing referral systems for higher-level care, may be an important step toward improving maternal outcomes at scale. In addition, given Nigeria's reliance on task-shifting strategies, investments in midwife-led emergency obstetric care are particularly critical (78, 79). The strong acceptance of bundled interventions such as E-MOTIVE and AMTSL underscores the importance of structured, repeated training and mentorship. For example, evidence from clinical observations indicated high adoption rates but variability and inconsistency in the application of AMTSL components across facilities, including irregular use of prophylactic uterotonics, inconsistent controlled cord traction, and absence of uterine massage protocols (34). However, in addition to increased adoption, fidelity to established care protocols was observed following structured training in the E-MOTIVE and AMTSL (54), suggesting that institutionalizing standardized training curricula, including refresher training and supportive supervision, may support fidelity and sustained implementation. Embedding implementation science metrics, including sustainability, penetration, equity, and patient-centeredness, into program evaluations of clinical effectiveness evaluation frameworks may help support effective implementation and scalability beyond controlled trial settings (80).

Logistical considerations should guide intervention selection. Heat-stable uterotonics (misoprostol, carbetocin), reusable devices (NASG), and simplified treatment algorithms may offer more sustainable solutions than resource-intensive combination therapies in resource-limited settings. Additionally, while this review did not directly evaluate antenatal care interventions, these findings suggest that strengthening antenatal anemia screening, iron supplementation, and nutritional programs may represent an important upstream strategy for PPH risk reduction.

Finally, funding agencies and national stakeholders should prioritize research that evaluates long-term sustainability, equity impacts, and women's lived experiences of PPH interventions. This ensures that the evidence is extended to include quality and cost, key components for improving health systems and patient experience, and ultimately important outcomes such as PPH risk and maternal mortality. Expanding implementation research in underrepresented regions, particularly North-Eastern Nigeria, is likely to be important for reducing geographic disparities in maternal mortality, though the current evidence base does not permit direct conclusions about intervention effectiveness in those settings.

### 4.2 Strengths and limitations

This systematic review has several strengths. First, it provides a comprehensive synthesis of pharmacological, device-based, and healthcare systems-level interventions for PPH within the Nigerian context, integrating both effectiveness and implementation perspectives. By applying Proctor et al.'s implementation outcomes framework, this review extends beyond traditional clinical effectiveness-focused syntheses and highlights important gaps in feasibility, adoption, and appropriateness. Second, the inclusion of multiple study designs, including randomized controlled trials, observational studies, mixed-methods studies, and qualitative research, allowed for a broad understanding of both the clinical and health system dimensions of PPH management. Third, the use of structured risk-of-bias assessment tools enhanced methodological rigor and transparency in evaluating study quality. Finally, the nascent approach to mapping provider and patient perspectives revealed the significant gaps.

Several limitations should be considered. The majority of included studies were conducted in tertiary care facilities, limiting generalizability to primary care settings where many Nigerian women receive care. Implementation outcomes were inconsistently defined and infrequently reported, restricting comparative analysis across studies. Heterogeneity in intervention types and intervention intent, outcome definitions, and study designs precluded meta-analysis and limited the ability to quantify pooled effects or draw direct comparisons across studies. Readers are cautioned against drawing equivalences across distinct clinical populations and intervention contexts. Additionally, publication bias cannot be excluded, as unpublished program evaluations, articles from reference list screening or citation searching, and grey literature were not included. The decision to exclude articles not indexed in the searched databases was made in order to maintain a manageable and reproducible search scope within the resources available to the team. Nonetheless, the searched databases encompass a broad and well-established collection of peer-reviewed literature with a measurable and replicable evidence base, providing confidence that the most rigorously evaluated clinical and implementation evidence on PPH interventions in Nigeria was captured. Finally, reliance on reported data may have resulted in an underestimation of implementation processes that were conducted but not explicitly described. Despite these limitations, the review provides important insights to guide policy, implementation, and future research priorities.

### 4.3 Conclusion

This review highlights the variety of evidence-based clinical interventions for PPH prevention and management, with opportunities to further evaluate successful implementation in different contexts. Based on the available evidence, bundled care approaches, structured workforce training, and context-appropriate pharmacologic and device-based interventions alongside context-appropriate pharmacologic and device-based interventions, represent the most promising foundation for strengthening PPH prevention and management within the Nigerian health system. Policymakers should consider prioritizing these approaches while simultaneously investing to strengthen primary-level care infrastructure, standardizing training, improving supply-chain reliability, implementing maternal anemia prevention strategies, and embedding implementation science evaluation into maternal health programs. Instituting these upstream strategies will increase the likelihood of achieving meaningful and equitable progress in eliminating PPH-related mortality. Persistent gaps, including limited primary-level research, underrepresentation of high-burden regions, and inadequate reporting of implementation and patient-centered outcomes, constrain translation of clinical effectiveness into sustained reductions in maternal mortality. Researchers should prioritize studies that examine sustainability, equity, and women's perspectives while expanding evidence generation in underserved regions. Only through integrated clinical and systems-level interventions can sustained reductions in PPH-related mortality be realized in Nigeria and comparable low-resource settings.

## Data Availability

The original contributions presented in the study are included in the article/Supplementary Material, further inquiries can be directed to the corresponding author/s.
