# Supplementary material for: Clinical and healthcare systems-level interventions for postpartum hemorrhage prevention and management in Nigeria: a systematic review of effectiveness and implementation outcomes
Source: Front Health Serv. 2026 Aug 17;6:1886853. doi: 10.3389/frhs.2026.1886853 (PMC13526822; doi:10.3389/frhs.2026.1886853)
Supplement: Supplementary file 1 [file Supplementaryfile1.docx]

**Supplemental Table 1: Inter-Rater Reliability**

| **Title Abstract Screening: Inter-rater Reliability** | | | |  |  |  |  |  |  |  |
| --- | --- | --- | --- | --- | --- | --- | --- | --- | --- | --- |
| Reviewer A | Reviewer B | A Yes,  B Yes | A Yes,  B No | A No,  B Yes | A No,  B No | Proportionate Agreement | Yes Probability | No Probability | Random Agreement Probability | **Cohen's Kappa** |
| MU | PO | 76 | 54 | 40 | 363 | 0.82364 | 0.05308 | 0.59154 | 0.64463 | **0.50373** |
| MA | PO | 6 | 15 | 5 | 167 | 0.89637 | 0.0062 | 0.8404 | 0.8466 | **0.32447** |
| MA | MU | 1 | 1 | 4 | 4 | 0.5 | 0.1 | 0.4 | 0.5 | **0** |
| AA | MU | 1 | 1 | 0 | 1 | 0.66667 | 0.22222 | 0.22222 | 0.44444 | **0.4** |
|  |  |  |  |  |  |  |  |  |  |  |
| **Full Text Review: Inter-Rater Reliability** | | |  |  |  |  |  |  |  |  |
| Reviewer A | Reviewer B | A Include, B Include | A Include, B Exclude | A Exclude, B Include | A Exclude, B Exclude | Proportionate Agreement | Yes Probability | No Probability | Random Agreement Probability | **Cohen's Kappa** |
| MU | PO | 27 | 10 | 11 | 56 | 0.79808 | 0.12999 | 0.40884 | 0.53883 | **0.56215** |
| DV | PO | 0 | 0 | 0 | 2 | 1 | 0 | 1 | 1 | **NaN** |
| AA | MU | 0 | 0 | 0 | 2 | 1 | 0 | 1 | 1 | **NaN** |
| MU | SF | 1 | 0 | 2 | 5 | 0.75 | 0.04688 | 0.54688 | 0.59375 | **0.38462** |
| MA | MU | 0 | 1 | 2 | 0 | 0 | 0.22222 | 0.22222 | 0.44444 | **-0.8** |

**Supplementary Notes: Search Strategy**

Web of Science

TS=(Nigeria OR Nigerian OR Abia OR "Abia state" OR Adamawa OR "Adamawa state" OR "Akwa Ibom" OR "Akwa Ibom state" OR "Akwa-ibom" OR "Akwa-ibom state" OR "Akwa-Ibom" OR "Akwa-Ibom state" OR Anambra OR "Anambra state" OR Bauchi OR "Bauchi state" OR Bayelsa OR "Bayelsa state" OR Benue OR "Benue state" OR Borno OR "Borno state" OR "Cross river" OR "Cross river state" OR "Cross-river" OR "Cross-river state" OR "Cross-River" OR "Cross-River state" OR Delta OR "Delta state" OR Ebonyi OR "Ebonyi state" OR Edo OR "Edo state" OR Ekiti OR "Ekiti state" OR Enugu OR "Enugu state" OR Gombe OR "Gombe state" OR Imo OR "Imo state" OR Jigawa OR "Jigawa state" OR Kaduna OR "Kaduna state" OR Kano OR "Kano state" OR Katsina OR "Katsina state" OR Kebbi OR "Kebbi state" OR Kogi OR "Kogi state" OR Kwara OR "Kwara state" OR Lagos OR "Lagos state" OR Nasarawa OR "Nasarawa state" OR Niger OR "Niger state" OR Ogun OR "Ogun state" OR Ondo OR "Ondo state" OR Osun OR "Osun state" OR Oyo OR "Oyo state" OR Plateau OR "Plateau state" OR Rivers OR "Rivers state" OR Sokoto OR "Sokoto state" OR Taraba OR "Taraba state" OR Yobe OR "Yobe state" OR Zamfara OR "Zamfara state" OR "Federal Capital Territory" OR FCT OR Abuja) AND TS=("health provider uptake" OR "health provider acceptance" OR "health care utilization" OR "healthcare utilization" OR "patient acceptance of healthcare" OR "patient attitude" OR "patient compliance" OR "patient participation" OR "patient adherence" OR adoption OR adopt OR adopted OR adopts OR uptake OR utilization OR implementation OR "intention to try" OR appropriateness OR appropriate OR "program evaluation" OR "perceived fit" OR relevance OR compatibility OR suitability OR usefulness OR practicability OR "program effectiveness" OR cost OR costs OR "cost effectiveness" OR "cost benefit" OR "cost analysis" OR "cost analyses" OR affordability OR pricing OR "cost measures" OR "cost comparison" OR value OR expenditures OR feasibility OR feasible OR utility OR suitability OR practicability OR fidelity OR delivery OR adherence OR availability OR integrity OR quality OR "quality of medications" OR "quality of services" OR "quality of health services" OR "quality of medical service*" OR "quality of ante-natal services" OR "quality of antenatal service*" OR "quality of obstetric service*" OR reliability OR penetration OR institutionalization OR spread OR "service access" OR access OR accessible OR accessibility OR sustainability OR sustain OR sustainable OR "program evaluation" OR implementation OR "evidence-based practice" OR innovation OR scale OR assessment OR measurement OR "translation research" OR "scale-up" OR scalability) AND TS=(prevention OR "primary prevention" OR "primary disease prevention" OR "secondary prevention" OR "secondary disease prevention" OR "preventive management" OR management OR "preventive intervention*" OR intervention OR treatment* OR protocol OR guideline* OR "best practices" OR practices OR care OR strategy OR "obstetric service" OR "screening tool*" OR bundle* OR "obstetric hemorrhage bundle*" OR "OH bundle*" OR therap*) AND TS=("obstetrical hemorrhage" OR "obstetric hemorrhage" OR "obstetric haemorrhage" OR "postpartum haemorrhage" OR "post-partum haemorrhage" OR "postpartum hemorrhage" OR "vaginal bleeding" OR "uterine hemorrhage" OR "uterine bleeding" OR "antepartum hemorrhage" OR "intrapartum hemorrhage" OR "maternal bleeding" OR PPH OR "antenatal bleeding" OR "ante-natal bleeding" OR "post-natal bleeding" OR "postnatal bleeding")

PAIS

ti,ab(Nigeria OR Nigerian OR Abia OR "Abia state" OR Adamawa OR "Adamawa state" OR "Akwa Ibom" OR "Akwa Ibom state" OR "Akwa-ibom" OR "Akwa-ibom state" OR "Akwa-Ibom" OR "Akwa-Ibom state" OR Anambra OR "Anambra state" OR Bauchi OR "Bauchi state" OR Bayelsa OR "Bayelsa state" OR Benue OR "Benue state" OR Borno OR "Borno state" OR "Cross river" OR "Cross river state" OR "Cross-river" OR "Cross-river state" OR "Cross-River" OR "Cross-River state" OR Delta OR "Delta state" OR Ebonyi OR "Ebonyi state" OR Edo OR "Edo state" OR Ekiti OR "Ekiti state" OR Enugu OR "Enugu state" OR Gombe OR "Gombe state" OR Imo OR "Imo state" OR Jigawa OR "Jigawa state" OR Kaduna OR "Kaduna state" OR Kano OR "Kano state" OR Katsina OR "Katsina state" OR Kebbi OR "Kebbi state" OR Kogi OR "Kogi state" OR Kwara OR "Kwara state" OR Lagos OR "Lagos state" OR Nasarawa OR "Nasarawa state" OR Niger OR "Niger state" OR Ogun OR "Ogun state" OR Ondo OR "Ondo state" OR Osun OR "Osun state" OR Oyo OR "Oyo state" OR Plateau OR "Plateau state" OR Rivers OR "Rivers state" OR Sokoto OR "Sokoto state" OR Taraba OR "Taraba state" OR Yobe OR "Yobe state" OR Zamfara OR "Zamfara state" OR "Federal Capital Territory" OR FCT OR Abuja) AND ti,ab("health provider uptake" OR "health provider acceptance" OR "health care utilization" OR "healthcare utilization" OR "patient acceptance of healthcare" OR "patient attitude" OR "patient compliance" OR "patient participation" OR "patient adherence" OR adoption OR adopt OR adopted OR adopts OR uptake OR utilization OR implementation OR "intention to try" OR appropriateness OR appropriate OR "program evaluation" OR "perceived fit" OR relevance OR compatibility OR suitability OR usefulness OR practicability OR "program effectiveness" OR cost OR costs OR "cost effectiveness" OR "cost benefit" OR "cost analysis" OR "cost analyses" OR affordability OR pricing OR "cost measures" OR "cost comparison" OR value OR expenditures OR feasibility OR feasible OR utility OR suitability OR practicability OR fidelity OR delivery OR adherence OR availability OR integrity OR quality OR "quality of medications" OR "quality of services" OR "quality of health services" OR "quality of medical service*" OR "quality of ante-natal services" OR "quality of antenatal service*" OR "quality of obstetric service*" OR reliability OR penetration OR institutionalization OR spread OR "service access" OR access OR accessible OR accessibility OR sustainability OR sustain OR sustainable OR "program evaluation" OR implementation OR "evidence-based practice" OR innovation OR scale OR assessment OR measurement OR "translation research" OR "scale-up" OR scalability) AND ti,ab(prevention OR "primary prevention" OR "primary disease prevention" OR "secondary prevention" OR "secondary disease prevention" OR "preventive management" OR management OR "preventive intervention*" OR intervention OR treatment* OR protocol OR guideline* OR "best practices" OR practices OR care OR strategy OR "obstetric service" OR "screening tool*" OR bundle* OR "obstetric hemorrhage bundle*" OR "OH bundle*" OR therap*) AND ti,ab("obstetrical hemorrhage" OR "obstetric hemorrhage" OR "obstetric haemorrhage" OR "postpartum haemorrhage" OR "post-partum haemorrhage" OR "postpartum hemorrhage" OR "vaginal bleeding" OR "uterine hemorrhage" OR "uterine bleeding" OR "antepartum hemorrhage" OR "intrapartum hemorrhage" OR "maternal bleeding" OR PPH OR "antenatal bleeding" OR "ante-natal bleeding" OR "post-natal bleeding" OR "postnatal bleeding")

PUBMED

(Nigeria[tw] OR Nigerian[tw] OR Abia[tw] OR "Abia state"[tw] OR Adamawa[tw] OR "Adamawa state"[tw] OR "Akwa Ibom"[tw] OR "Akwa Ibom state"[tw] OR "Akwa-ibom"[tw] OR "Akwa-ibom state"[tw] OR "Akwa-Ibom"[tw] OR "Akwa-Ibom state"[tw] OR Anambra[tw] OR "Anambra state"[tw] OR Bauchi[tw] OR "Bauchi state"[tw] OR Bayelsa[tw] OR "Bayelsa state"[tw] OR Benue[tw] OR "Benue state"[tw] OR Borno[tw] OR "Borno state"[tw] OR "Cross river"[tw] OR "Cross river state"[tw] OR "Cross-river"[tw] OR "Cross-river state"[tw] OR "Cross-River"[tw] OR "Cross-River state"[tw] OR Delta[tw] OR "Delta state"[tw] OR Ebonyi[tw] OR "Ebonyi state"[tw] OR Edo[tw] OR "Edo state"[tw] OR Ekiti[tw] OR "Ekiti state"[tw] OR Enugu[tw] OR "Enugu state"[tw] OR Gombe[tw] OR "Gombe state"[tw] OR Imo[tw] OR "Imo state"[tw] OR Jigawa[tw] OR "Jigawa state"[tw] OR Kaduna[tw] OR "Kaduna state"[tw] OR Kano[tw] OR "Kano state"[tw] OR Katsina[tw] OR "Katsina state"[tw] OR Kebbi[tw] OR "Kebbi state"[tw] OR Kogi[tw] OR "Kogi state"[tw] OR Kwara[tw] OR "Kwara state"[tw] OR Lagos[tw] OR "Lagos state"[tw] OR Nasarawa[tw] OR "Nasarawa state"[tw] OR Niger[tw] OR "Niger state"[tw] OR Ogun[tw] OR "Ogun state"[tw] OR Ondo[tw] OR "Ondo state"[tw] OR Osun[tw] OR "Osun state"[tw] OR Oyo[tw] OR "Oyo state"[tw] OR Plateau[tw] OR "Plateau state"[tw] OR Rivers[tw] OR "Rivers state"[tw] OR Sokoto[tw] OR "Sokoto state"[tw] OR Taraba[tw] OR "Taraba state"[tw] OR Yobe[tw] OR "Yobe state"[tw] OR Zamfara[tw] OR "Zamfara state"[tw] OR "Federal Capital Territory"[tw] OR FCT[tw] OR Abuja[tw]) AND ("health provider uptake"[tw] OR "health provider acceptance"[tw] OR "health care utilization"[tw] OR "healthcare utilization"[tw] OR "patient acceptance of healthcare"[tw] OR "patient attitude"[tw] OR "patient compliance"[tw] OR "patient participation"[tw] OR "patient adherence"[tw] OR adoption[tw] OR adopt[tw] OR adopted[tw] OR adopts[tw] OR uptake[tw] OR utilization[tw] OR implementation[tw] OR "intention to try"[tw] OR appropriateness[tw] OR appropriate[tw] OR "program evaluation"[tw] OR "perceived fit"[tw] OR relevance[tw] OR compatibility[tw] OR suitability[tw] OR usefulness[tw] OR practicability[tw] OR "program effectiveness"[tw] OR cost[tw] OR costs[tw] OR "cost effectiveness"[tw] OR "cost benefit"[tw] OR "cost analysis"[tw] OR "cost analyses"[tw] OR affordability[tw] OR pricing[tw] OR "cost measures"[tw] OR "cost comparison"[tw] OR value[tw] OR expenditures[tw] OR feasibility[tw] OR feasible[tw] OR utility[tw] OR suitability[tw] OR practicability[tw] OR fidelity[tw] OR delivery[tw] OR adherence[tw] OR availability[tw] OR integrity[tw] OR quality[tw] OR "quality of medications"[tw] OR "quality of services"[tw] OR "quality of health services"[tw] OR "quality of medical service*"[tw] OR "quality of ante-natal services"[tw] OR "quality of antenatal service*"[tw] OR "quality of obstetric service*"[tw] OR reliability[tw] OR penetration[tw] OR institutionalization[tw] OR spread[tw] OR "service access"[tw] OR access[tw] OR accessible[tw] OR accessibility[tw] OR sustainability[tw] OR sustain[tw] OR sustainable[tw] OR "program evaluation"[tw] OR implementation[tw] OR "evidence-based practice"[tw] OR innovation[tw] OR scale[tw] OR assessment[tw] OR measurement[tw] OR "translation research"[tw] OR "scale-up"[tw] OR scalability[tw]) AND (prevention[tw] OR "primary prevention"[tw] OR "primary disease prevention"[tw] OR "secondary prevention"[tw] OR "secondary disease prevention"[tw] OR "preventive management"[tw] OR management[tw] OR "preventive intervention*"[tw] OR intervention[tw] OR treatment*[tw] OR protocol[tw] OR guideline*[tw] OR "best practices"[tw] OR practices[tw] OR care[tw] OR strategy[tw] OR "obstetric service"[tw] OR "screening tool*"[tw] OR bundle*[tw] OR "obstetric hemorrhage bundle*"[tw] OR "OH bundle*"[tw] OR therap*[tw]) AND ("obstetrical hemorrhage"[tw] OR "obstetric hemorrhage"[tw] OR "obstetric haemorrhage"[tw] OR "postpartum haemorrhage"[tw] OR "post-partum haemorrhage"[tw] OR "postpartum hemorrhage"[tw] OR "vaginal bleeding"[tw] OR "uterine hemorrhage"[tw] OR "uterine bleeding"[tw] OR "antepartum hemorrhage"[tw] OR "intrapartum hemorrhage"[tw] OR "maternal bleeding"[tw] OR PPH[tw] OR "antenatal bleeding"[tw] OR "ante-natal bleeding"[tw] OR "post-natal bleeding"[tw] OR "postnatal bleeding"[tw])
